# Reduction of the Cycle Time in the Biopsies Diagnosis Through a Simulation Based on the Box Müller Algorithm

**DOI:** 10.3389/fpubh.2022.809534

**Published:** 2022-04-04

**Authors:** Félix Badilla-Murillo, Bernal Vargas-Vargas, Oscar Víquez-Acuña, Justo García-Sanz-Calcedo

**Affiliations:** ^1^Industrial Production Engineering, Instituto Tecnológico de Costa Rica, Cartago, Costa Rica; ^2^Computer Engineering, Instituto Tecnológico de Costa Rica, Cartago, Costa Rica; ^3^Engineering Projects Area, University de Extremadura, Badajoz, Spain

**Keywords:** discrete event simulation, hospital management, process model, installed productive capacity, healthcare engineering, Box Müller algorithm, logistics (business)

## Abstract

Anatomic pathology services study disease in hospitals on the basis of macroscopic and microscopic examination of organs and tissues. The focus of this research investigation was on improving clinical biopsy diagnosis times through simulation based on the Box-Muller algorithm to reduce the waiting time in the diagnosis of clinical biopsies. The data were provided by a hospital in San José (Costa Rica). They covered 5 years and showed waiting times for a pathological diagnosis that for some biopsies were close to 120 days. The correlation between the main causes identified and the cycle time in the biopsy diagnostic process was defined. A statistical analysis of the variables most representative of the process and of the waiting times was carried out. It followed the DMAIC structure (Define, Measure, Analyse, Improve, Control) for the continuous improvement of processes. Two of the activities of the process were identified as being the main bottlenecks. Their processing times had a normal distribution, for which reason a Box-Muller algorithm was used to generate the simulation model. The results showed that waiting times for a diagnosis can be reduced to 3 days, for a productive capacity of 8 000 biopsies per annum, optimizing the logistics performance of health care.

## Introduction

Discrete Event Simulation (DES) is a technique used to analyse the installed productive capacity in a process. In health services, it can be applied to determine the number of nursing professionals required to meet a certain demand (1, 2). There is today a considerable variety of applications that allow the development of simulation models in health services, as is the case of the creation of algorithms using software (3). Most health care simulation models are constructed using DES, system dynamics, or agent-based modeling (4).

Simulation models can be developed to mimic the complex functioning of health services. Examples are how the patient's journey or a change in the number of emergency department physicians influence the performance of an emergency ward or an angiography room (5, 6). DES models can also be used to determine an energy efficient route by defining candidate routes and taking into account the state of the network to finally select the most efficient route among the alternatives proposed (7). There has been work (8) integrating DES with Lean Management (LM) and Complexity Scoring (CS) to propose a method for analyzing the problems associated with lean manufacturing. DES models make it possible to investigate the relationship between a supply chain's configuration and performance in the context of a modular construction project (9).

In addition, DES enables clinical decision-making in patient care to be improved in resolving real health system problems. Hybrid Simulation (HS) uses at least two different simulation modalities, so that their combination, with an appropriate alignment, coordination, and interface between them, will let one of them improve the other (10). The key indicators affecting the privacy of Big Data in health management have been identified (11). A fuzzy theory-based model of risk access control which was then used for Big Data management in intelligent medical treatment was established. The problem of inaccurate experimental results due to the lack of real data when dealing with real problems was also resolved.

The importance of hospital performance management is recognized for providing effective quality of care and is a key in any healthcare organization. Furthermore, it closes the gap between conceptual planning of organizational goals and physical monitoring of the status of daily operations (12). In the operational management of hospitals, a reliability focused approach to maintenance has been combined with evolutionary optimization to develop optimal maintenance plans for hospital facilities in four areas: intensive care units, emergency rooms, operating theaters, and patient rooms (13, 14).

An important input in simulation models is that of the algorithms that generate random numbers with a given probability distribution (15). Among them, one might mention the krill herd (KH) algorithm which is inspired by the grazing behavior of krill swarms. The objective function in the KH optimization process is based on the minimum distance between the location of the food and the position of a krill individual (16). Another is quasi-Monte Carlo simulation. This has been growing in popularity due to its convergence rate and the existence of simple statistical tools to analyse the error of its estimates. One such tool is the Box-Muller transform which takes as input two uniformly distributed random numbers and returns two independent pseudo-random numbers from the standard normal distribution (17).

The Box-Muller algorithm is also used for experimental modeling in the stochastic analysis of variables with a Maxwell-Boltzmann distribution, as well as the Ornstein-Uhlenbeck and Einstein approximations (18). Another application is for quantum measurement experiments in which symmetry breaking is used to enhance a microscopic signal. The quantum system comprises a system-apparatus-environment and uses the Box-Muller algorithm to generate random numbers with a standard normal distribution (19). Monte-Carlo simulation has been used to analyse the concentration of dust particles on the surface of a photovoltaic cell, with a dust particle being assumed to be spherical in shape with a radius that depends on the log-normal distribution function implemented by means of the transformation to the normal distribution using the Box-Muller algorithm (20). In this present study, the Box-Muller algorithm is used to generate the random numbers of the pathology department because it was identified that they have a normal probability distribution, and in this way variability and uncertainty are incorporated into the analysis. Pathologic reports can confirm or exclude the presence of particular diseases, such as the rule of microbial agents in an infection or check cancers (21–24).

The problem of the Department of Pathology of Costa Rica is focused on its lack of operational efficiency. The application of the Box Müller algorithm is intended to improve the operational performance of the hospital, thus bridging the current technological knowledge gap.

The objective of this work was to develop a simulation model based on the Box-Muller algorithm to reduce the waiting time in the diagnosis of clinical biopsies. This will allow the health service's installed productive capacity to be analyzed by identifying the critical factors that most affect it. It will allow also to be put forward solutions that optimize the logistics performance of health care.

## Materials and Methods

The data analyzed correspond to a 5-year period in a hospital in San José (Costa Rica), with average waiting times for a pathological diagnosis close to 120 days. An application was designed in Visual Basic for Applications (VBA) capable of making discrete event simulation models based on the Box-Muller algorithm to generate random numbers with a normal probability distribution. A graphical interface with forms was also created.

The process of biopsy diagnosis in the health service has six stages:

1. Collection of tissue specimens that need to be analyzed.
2. Cutting the specimens depending on the type of organ and its physical characteristics.
3. Embedding the biopsy specimens in paraffin wax blocks.
4. Trimming and preparation of glass slides.
5. Reading and diagnosis of the slides by the pathologist.
6. Delivery of report with results.

To identify bottlenecks in the process, these stages were broken down into 36 activities categorized as operations, inspections, storage, waiting, or transport. The most representative elements for the analysis of the anatomic pathology department's installed productive capacity are the different work areas, their working hours, the staff assigned to each, and its equipment. Table 1 shows the resources available in the anatomic pathology department.

**Table 1:** Resources available in the anatomic pathology department.

**Area**	**Shifts**	**Staff**	**Equipment**
An autopsy room	06:00–14:00 14:00–22:00	**Two technicians**. After 22:00, the area remains closed and unstaffed. This is the section of the department that stays open longest.	Two cooling chambers of two modules each Crane for cadavers Camera Dissection table Computer Bright screen Scale
A biopsy cutting room	07:00–16:00	**One technician**	A computer for data entry into SIPAT Cutting instruments (blades, etc.)
A histology room	07:00–16:00	**Two technicians** work in this room, both on the 07:00–16:00 shift	A cryostat for frozen biopsies, and a refrigerator to store specimens Two automatic tissue processors A microwave oven for special stains A laboratory oven Slide dryer
A cytology room	07:00–16:00		The cytology output is quite low. Also, due to the quantity of biopsies to be analyzed, this place has been picked to use for the storage of biopsies awaiting analysis by the pathologist.
Three clinics (offices) for pathologists	07:00–16:00	**One pathologist** doing administrative and operational activities	Each office is equipped with a computer and a microscope
A meeting room	07:00–16:00		Desks Board Microscopes

Between January 2015 and October 2017, the hospital had three pathologists at that time so that the throughput of biopsies remained stable. In addition, for that period, it was possible to appreciate how not only was attention given provided to that hospital, but support was provided to the health service network very equitably.

In November 2017, the throughput of the medical center dropped significantly due to the departure of the team of pathologists. That center temporarily sent biopsies for analysis to another hospital in the health services network. Subsequently, the department has a staff doctor who combines administrative tasks as head of the area with the operations of biopsy analyses. In addition, pathologists provide support through overtime, but this support is external to the hospital and sporadic.

**Figure 1:**
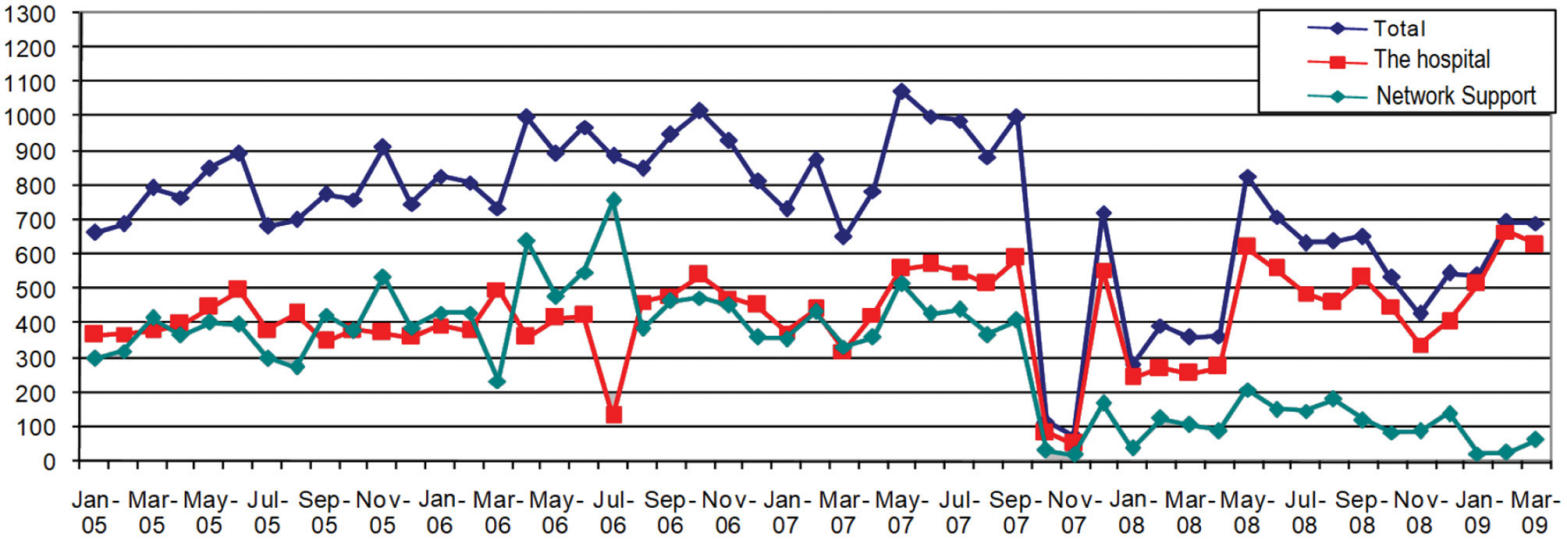
Biopsies performed in the pathology department. Source: Anatomic Pathology Service.

As shown in Figure 1, in order to meet the demand for the service, support to the network was stopped due to the limited number of specialized personnel. Nonetheless, the demand shows a tendency to increase, negatively impacting even more the output of support to the network. The users who receive this service are affected by having to perform their biopsies at other centers. In absorbing the unsatisfied output, this in turn saturates the capacity of the hospitals that provide this service.

### Box-Muller Transform

Box and Muller suggested the following model of mathematical statistics used to generate random numbers with a normal distribution. If *U*_1_ and *U*_2_ are independent uniformly distributed variables in the interval (0,1), then $x_{1}=\sqrt{-2\ln\;U_{1}}\sin2\pi U_{2}$, is precisely the standard Gaussian N(0,1) (25, 26). Using this method, an independent sequence N(μ,σ^2^) of random variables is taken:


(1) $$\{\begin{array}{c} \mu +\sigma x_{1},\;x_{1}=\sqrt{-2\ln U_{1}}\;\sin2\pi \;U_{2}\\ \mu +\sigma x_{2},\;x_{2}=\sqrt{-2\ln U_{3}}\;\sin2\pi 4\\ \mu +\sigma x_{3},\;x_{3}=\sqrt{-2\ln U_{5}}\;\sin2\pi \;U_{6} \end{array}$$

This method is relatively fast and provides normally distributed random numbers.

### The DMAIC Structure

This research was based on the continuous improvement stages of DMAIC (Define, Measure, Analyse, Improve, Control) processes, which is part of the six-sigma set of production strategies (27–29). The following paragraphs will set out the approach taken to each of the stages.

#### Define Stage

The variability problem to be dealt with in the study (27) was established. It corresponds to the current cycle time of biopsy diagnoses. This was considered to be long since it can be up to 120 days. There would be a consequent significant deterioration of the patient's health, and hence greater costs borne by the healthcare system. The objective is to reduce the waiting time for the diagnosis of biopsies by designing a DES model.

#### Measure Stage

A data collection plan was determined for the variables required for the simulation model. For this, the official information sources (30) were validated. In the case of the cycle time data, it was necessary to determine the type of their probability distribution by applying goodness-of-fit tests in accordance with Equation 2:


(2) $$\begin{array}{r} \chi^{2}=\overset{k}{\underset{i=1}{\sum }}\frac{{\left(o_{i}-e_{i}\right)}^{2}}{e_{i}} \end{array}$$

where χ^2^ is the value of a random variable whose sampling distribution approximates the chi-squared distribution with *v* = *k*−1 degrees of freedom, where *k* is the total number of data comprising the sample. The variables *o*_*i*_ and *e*_*i*_ represent the *i-th* cell observed and expected frequencies, respectively. This test was applied to determine whether the data are described by a normal probability distribution, validating it with a *p*-value > 0.05 significance (α) used and an AD (Anderson-Darling) statistic close to zero. The tool used for the analysis was MINITAB^®^ 19.2 (2020). Table 2 presents the collection plan.

**Table 2:** The study's data collection plan.

**Variable**	**Description**	**Type**	**Source**
Biopsies received	Biopsies that were received by the anatomic pathology department	Quantitative; discrete	Hospital's software
Biopsies with diagnosis	Biopsies that were analyzed by the pathologist, and have a report and diagnosis	Quantitative; discrete	Hospital's software
Cycle time for biopsy review (*μ_1_*)	Mean duration of biopsy review by the pathologist, i.e., from their receiving a slide until making a diagnosis $\mu_{1}=\frac{\overset{n}{\underset{i=0}{\sum }}s_{i}}{n}\;$ *s* = cycle time for biopsy *i* *n* = total biopsies analyzed by the pathologist	Quantitative; continuous	Hospital's software
Cycle time for biopsy cutting (*μ_2_*)	Mean duration of the technician's biopsy cutting process $\mu_{2}=\frac{\overset{n}{\underset{i=0}{\sum }}s_{i}}{n}\;$ *s* = cycle time for cutting biopsy *i* *n* = total biopsies analyzed by the technician	Quantitative; continuous	Hospital's software
Time between biopsy arrivals (λ)	Mean time between biopsy arrivals $\lambda =\frac{\overset{n}{\underset{i=0}{\sum }}t_{i}}{n}\;$ t = time between arrivals for biopsy *i* n = total biopsies arriving at the department	Quantitative; continuous	Hospital's software
Activity	Activities or tasks that make up the biopsy analysis process in the anatomic pathology department	Qualitative; nominal	Process
Type of activity	Classification of activities such as operations, transportation, storage, waiting, and inspections	Qualitative; nominal	Process
Patient demand	The number of patients requiring the department's services in a year (summing those attended to, those in the queue, and those canceled)	Quantitative; discrete	Hospital's software
Mean waiting time	Mean waiting time in the system and the queue	Quantitative; continuous	Simulation model
Mean number of biopsies in the queue	Mean number of biopsies awaiting processing	Quantitative; discrete	Simulation model
Utilization	The ratio λ/μ of the time between arrivals to the cycle time	Quantitative; continuous	Simulation model

#### Analyze Stage

The correlation between the main causes identified and the cycle time in the biopsy diagnostic process was defined. This was done through the design of a VBA that considers these variables and the algorithms required to construct the pathology service simulation model. The installed productive capacity analysis is one of the most relevant aspects considered in this study. It lets one determine the bottleneck in the process, the use of resources, and the impact on the throughput. As part of the process, it was necessary to validate statistically the simulation model created against the real system using confidence intervals (31). To this end, the 1-month throughput data (*X*) were compared with the simulation data (*Y*) for the same period. If the interval created from this data set contains zero, it indicates that there is sufficient evidence to not reject the null hypothesis that there are no differences. Otherwise, there are differences.


(3) $$\begin{array}{r} H_{0}:X=Y \end{array}$$

(4) $$\begin{array}{r} H_{a}:X\neq Y \end{array}$$

(5) $$\left((X-Y)-t_{\upsilon -1,\;1-\alpha }^{*}\sqrt{\sigma_{X-Y}^{2}},(X-Y)+t_{\upsilon -1,\;1-\alpha }^{*}\sqrt{\sigma_{X-Y}^{2}}\right)$$

where *H*_0_ is the null hypothesis, *H*_*a*_ is the alternative hypothesis, the variable *X* represents the data obtained from the real system, *Y* corresponds to the data obtained through the simulation model, *X* the average of the real system data, *Y* the average of the simulation model data, t the statistic of a probability distribution t for ν-1 degrees of freedom and a 1-α degree of confidence, $\sigma_{X-Y}^{2}$ is the variance of the difference of the real system data with respect to the simulation model data.

#### Improve Stage

Different what-if scenarios were defined considering the results provided by the simulation model (32, 33). It is sought with them to match the installed productive capacity with the current demand for the department by making variations mainly to the amount of resources available (34).

#### Control Stage

The purpose of this phase is the transition from the improvement to the original process. For this, a set of indicators was developed with which to monitor the performance of the process, and thus determine whether the study's objective had been met (35, 36).

#### Economic Evaluation

In order to assess the cost-effectiveness of the measures implemented, an analysis was carried out including the following variables: annual fixed cost (US$), overtime (US$), total cost (US$), adjustment time (months), annual output (units), diagnosis (days) and cost per biopsy (US$).

## Results

The results of the study are detailed in the following subsections.

### Description of the Biopsy Diagnosis Process

**Figure 2:**
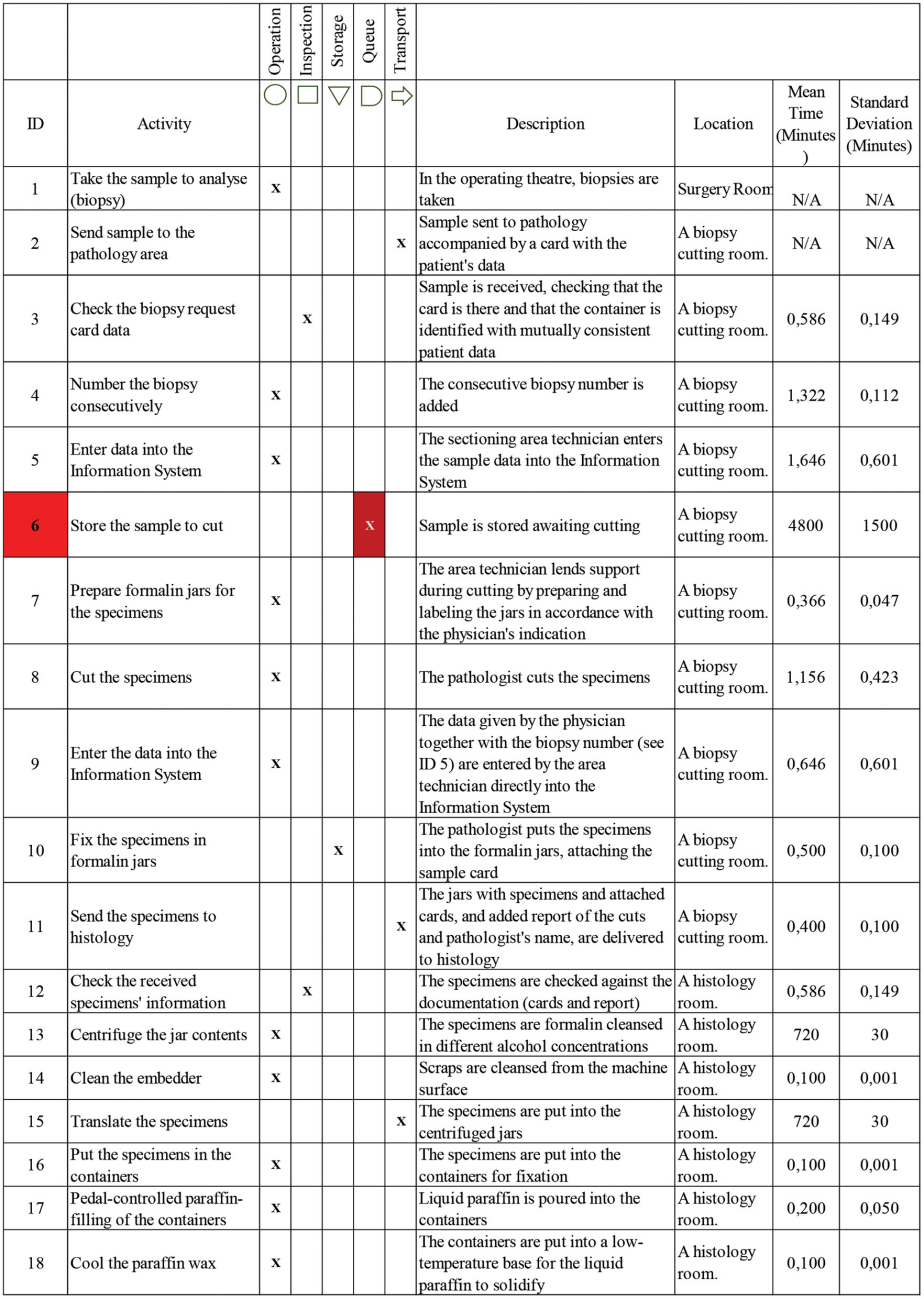
Flowchart of the biopsy process.

In the process, activities 6 and 32 stand out for their low times corresponding to waits that add no value to the process. Figure 2 shows the classification of the activities, including a description of each, the location where it takes place, the mean duration and standard deviation of the cycle times. The cycle time data for the 36 activities identified were obtained through sampling and data of the transactional information system of the anatomic pathology department.

### Comparative Analysis of the Biopsies Received With the Biopsies Diagnosed

The anatomic pathology department is responsible for making the diagnoses corresponding to biopsies, cytology, and autopsies. Biopsies account for 74.46% of the hospital's Anatomic Pathology Services output, cytology 25.09%, and autopsies 0.44%. For this reason, the analysis of biopsies is prioritized to determine how waiting times could be reduced for patients.

**Figure 3:**
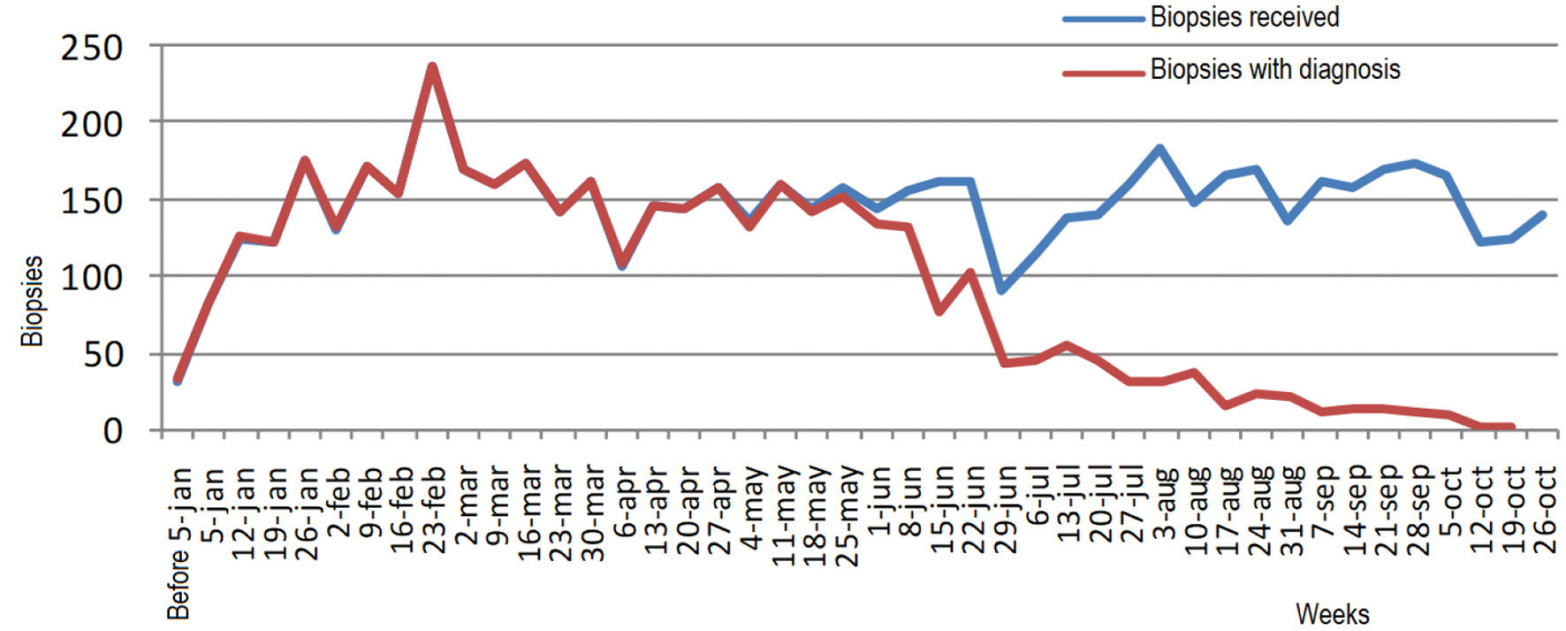
Relationship of the gap between biopsies received and biopsies diagnosed from the week they enter the anatomic pathology department.

Figure 3 shows the relationship of the gap between the biopsies received and the biopsies diagnosed from the week they enter the pathology department. The lag between the two starts from week 24. Thus, as the most recent date is approached and the gap between biopsies diagnosed and biopsies received grows there are biopsies of 4 months without analysis. This is because the hospital's throughput capacity cannot meet the demand.

### Statistical Analysis for Biopsies Received

Analysis of the data of the biopsies received weekly by the anatomic pathology department showed the demand to be normally distributed. In particular, the Anderson-Darling test yielded a *p*-value of 0.122, which is >0.05 significance used, and an *A*^2^ close to zero (0.58). The mean weekly demand for biopsies in the center was ~151.55 biopsies, with a standard deviation of 18.45. Thus, the 95% confidence interval of the mean is from 145.65 to 157.45, and of the standard deviation from 15.11 to 23.69. It is important to observe the data's distribution since one will be working with averages and deviations.

### Statistical Analysis for the Biopsy Response Time

Analysis of the time that elapses for a diagnosis after a biopsy entered the anatomic pathology department showed the average to be 123.57 days. The scatter of the data is considerable, the range being from 4 to 246 days. The data are not normally distributed as the Anderson-Darling test yielded a *p*-value below 0.05.

### Provenance of the Biopsies

**Figure 4:**
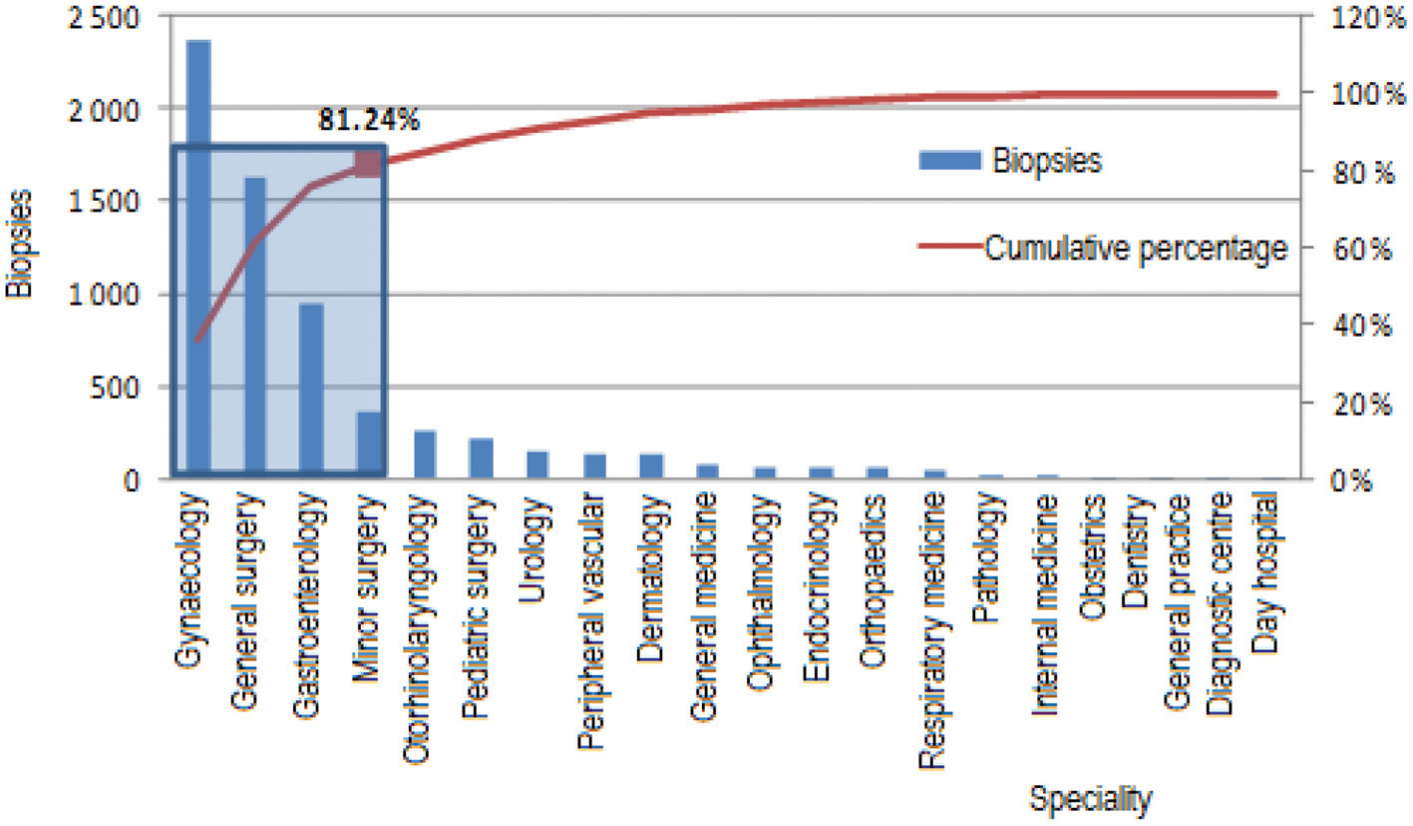
Pareto chart for the number of biopsies according to specialty.

Of the biopsies performed by the Anatomic Pathology Service, 87% corresponded to requests originating in the hospital itself, and the remaining 13% corresponded to the service provided to other hospitals of the Health Services Network. Of the total biopsies received in the *San Rafael de Alajuela Hospital*, 81.24% are from the specialities of gynecology, general surgery, gastroenterology, and minor surgery. The rest of the specialities account for the remaining 18.76%, as shown in Figure 4.

### Statistical Analysis of the Biopsy Process Turnaround Times

**Figure 5:**
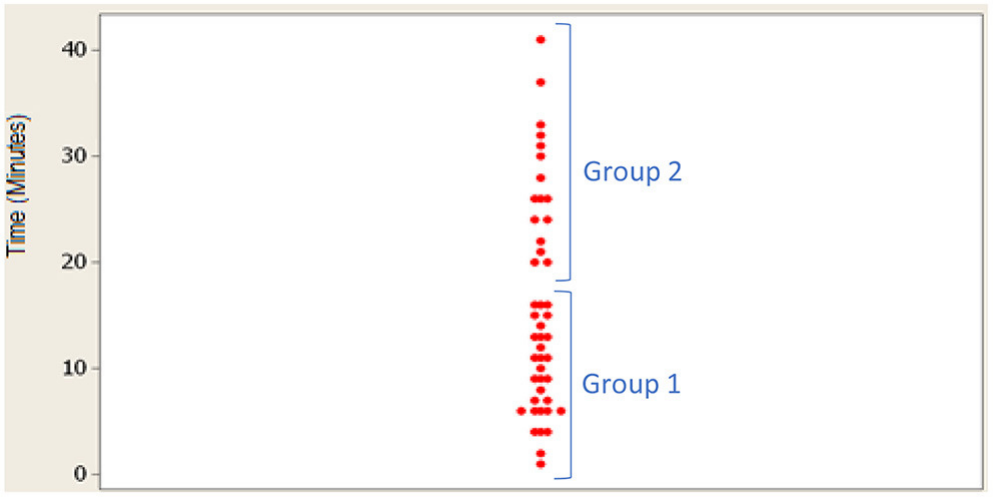
Plot of individual values of the time spent by the pathologist to examine a slide (activity 34 of the biopsy operations flowchart).

Biopsies represent 97% of the output of the pathology department. Ideally in a production process, throughput times would be normally distributed because this ensures a more stable and predictable output, reducing the expected variation (37). One of the activities most representative of the service corresponds to the time dedicated by the pathologist to reviewing the slides and making a diagnosis. Analysis of the summary of statistics of the data obtained in this activity reflected no greater significance, and the hypothesis of the Anderson-Darling test was rejected since *p* < 0.05 and *A*^2^ = 1.56. As the normal distribution could be rejected, the individual values were plotted to determine whether there might be some important trait to orient the analysis (Figure 5). It showed that the data could be classified into two groups: group 1 corresponding to times shorter than 16 min, and group 2 to times longer than 20 min.

This bimodal characteristic clearly reflects two different events within the pathologist's analysis of the biopsies, suggesting that there are two types of medical analysis, at least in time, that merit descriptive study. In group 1, the mean time of analysis by the pathologist is 9.26 min with a standard deviation of 4.30 min. In group 2, the mean is 27.39 min, and the standard deviation is 6.0 min. The data of both groups have a normal distribution. The results of the normality test indicated a *p-*value of 0.495 for group 1 and 0.407 for group 2; therefore, the *H*_0_ hypothesis was accepted.

According to the verification statistic, the null hypothesis of 24 min per biopsy is rejected and the alternative hypothesis is accepted, which indicates that the time to diagnose a biopsy is shorter, with an average of 16.40 min. At a 95% confidence level, the confidence interval of the times would be from 13.1 to 19.7 min per biopsy. For the lower limit of the interval, it can be inferred that a professional could analyse ~5 biopsies per h, and for the upper limit, 3.5 per h. In sum, the estimator of 2.5, which is the approximate rate which pathologists currently work with to define overtime is slower than the average yielded by the data.

It is important to note that the proportion with which these cases occur is 2:1 because for every two biopsies in group 1 there is one in group 2. This is equivalent to a third of the biopsy. This indicates that from group 1 a throughput of ~6 biopsies per h can be expected, and from group 2 ~2 biopsies per h.

### Sample Size

By means of a retrospective analysis, the power of the test was used to determine whether the sample size was adequate. The power corresponds to the probability of correctly rejecting *H*_0_ when it is false. In the group 1 it was found that the adequate sample size for a power of 0.95 and a standard deviation of 4.30 was 29. Therefore, it can be said that there is no significant difference >3, and it is unnecessary to increase the sample size. In the case of group 2, it was found that the adequate sample size for a power of 0.95 and a standard deviation of 6.001 is 21. In the real data analysis, the size of the sample taken was 18, which corresponds to a power of 0.90. This represents there being no significant difference >5, and it is unnecessary to have a greater sample size.

It is important to validate the sample size for the proportion of group 1 and group 2 biopsy diagnoses, since the data provided show a proportion of 0.33 and 0.67, respectively. With these parameters, the size of the sample analyzed of 54 corresponds to a power of 0.95. It is therefore unnecessary to increase the sample size to identify a significant difference between the proportions.

### Cutting of Biopsies by the Pathologist

Another important activity in which the pathologist is required is cutting the specimens. Although the normality hypothesis was rejected, the shape of the histogram showed some similarity to such a distribution. The mean of the data was 1.16 min with a standard deviation of 0.423. Another group was identified with a mean of 30 min and a standard deviation of 10 min. This corresponds to biopsy sections of organs or large segments of its, that account for 9% of the total cuts made.

### Percentage of Biopsies

A significant percentage of biopsies have not been diagnosed. The reason for this situation requires analysis. Of the biopsies received in the anatomic pathology department, 38% remain undiagnosed, corresponding to 2,468 of the 6,507 received up to October. The case is similar with the cytologies, with 32% reported without due diagnosis, corresponding to 72 of the 202 biopsies registered in the hospital.

#### Installed Productive Capacity of the Specialist Physician (Pathologist)

To analyse the capacity of the biopsy process, the throughput is defined considering the availability of the pathologist. This currently represents the bottleneck in the process. The activities in which this physician participates in the 1,776 h of work per year were simulated, excluding Saturdays, Sundays, holidays, vacations, and others. The proportions of the times to simulate were determined according to the combinations of biopsy cutting and analysis detailed in Table 3.

**Table 3:** Proportion of hours to simulate for the combination of biopsy cutting and analysis.

**ID**	**Activity**	**Mean time**	**SD**	**Cases**	**Proportion**
8	Specimen cutting Group 1	1.1563	0.423	30	90.9%
8	Specimen cutting Group 2	30	10	3	9.1%
34	Biopsy analysis Group 1	9.2571	4.3069	35	64.8%
34	Biopsy analysis Group 2	27.389	6.001	19	35.2%

The model was validated by comparing the biopsies performed with those produced using the interval analysis, obtaining (−115.3, 89.0). Thus, for activity 8 of the biopsy process, of the 33 cases of cutting by the pathologist, 30 (90.9%) belonged to cut 1 (C1), which has a mean throughput time of 1.1563 min with a standard deviation of 0.423 min. The remaining 9% of the cuts, denoted cut 2 (C2), took a mean of 30 min with a standard deviation of 10 min. For the biopsy analyses (activity 34), other different events were identified. Of the 54 cases of analysis by the pathologist, 35 (64.8%) belonged to analysis 1 (A1), which has a mean throughput time of 9.26 min with a standard deviation of 4.31 min. The remaining 35.2% of the analyses, denoted analysis 2 (A2), took a mean 27.39 min with a standard deviation of 6.00 min. Hence, four simulations (of 10 replicates each) were made applying the designed model to the possible combinations of cuts and analyses.

Simulation C1A1 (cut 1—analysis 1) resulted in a time of 1,176 h, simulation C1A2 591.71 h, simulation C2A1 137.07 h, and finally simulation C2A2 107.95 h. In total, as shown in Table 4, simulating these two activities requires 2,012.78 productive h from pathologists.

**Table 4:** Summary of simulated hours for the combination of possible biopsy cutting and analysis.

**Operation**	**Sim. C1A1**	**Sim. C1A2**	**Sim. C2A1**	**Sim. C2A2**	**Total hours**
Cutting (hours)	130.11	23.94	104.72	56.71	315.47
Analysis (hours)	1,045.95	567.77	32.35	51.24	1,697.31
Total (hours)	1,176.05	591.71	137.07	107.95	2,012.78

It was found, considering the information provided by the simulation model, that for 2,012 h it is possible to produce a total of 8,312 biopsies cut and analyzed. Another important factor to consider is the current demand of the pathology service, for which the current conditions were also simulated. To simulate this demand, random numbers were taken from a normal distribution with the mean of 151.55 biopsies per week and standard deviation of 18.45 observed in the demand for biopsies. The proposed annual output of 8,300 biopsies is capable of meeting the demand for 7 576 biopsies under current conditions, as shown in Table 5. Table 6 shows the number of pathologists required to meet current demand.

**Table 5:** Simulated biopsy output.

**Operation**	**Sim. C1A1**	**Sim. C1A2**	**Sim. C2A1**	**Sim. C2A2**	**Total hours**
Simulated output	6.748	1.242	210	112	8.312
Simulated demand	-	-	-	-	7.576

**Table 6:** Number of pathologists required to meet current demand.

**Human resources**	**Pathologists**	**Equivalent hours**	**Hours relative to demand**
Required human resources	1.13	2.013	0.00
Current human resources	0.75	1.332	−680.78
Proposed human resources	1.75	3.108	1,095.22

#### Simulation of the Biopsy Process

Once the number of pathologists needed to meet the demand for the biopsy process had been identified, the interaction of all activities was analyzed. The simulation was carried out with five replicates of 40 h (1 week), eliminating the non-value-aggregating storage activities (6 and 32). With the simulation, the model indicated that an average 239 biopsies can be generated weekly, more than the 151 weekly demanded by the service's users.

With respect to the activities' aggregate values, the time dedicated to operations is foremost with 108 h. Nonetheless, storage and transportation account for 85 h between them. These types of activities should therefore be reduced for future studies and to further refine the process.

Activities with more than 90% of use were identified. These were activity 19, storing the blocks in the refrigerators, activity 33, transferring the blocks to be analyzed, and activity 34, diagnosis of the slides by the pathologist. The first two are irrelevant since the method can be adapted by varying the quantity of blocks, but the pathologist's diagnosis will be the activity that sets the pattern for the entire production process, as shown in Table 7.

**Table 7:** Forty-hour simulation of the pathology department's biopsy process.

**Proceso:**	**Biopsy processing (Group 1)**								**Hours**	**Process inv. (units)**
**Cod Proc:**	**9,000**							**Operations**	**108.04**	**4,780**	
**Empresa:**	**Hospital**							**Inspections**	**4.676**	**478**	
**Cod Emp:**	**20**							**Transport**	**43.866**	**1,195**	
**Replicas:**	**5**							**Queue**	**19.968**	**239**	
**Simulacion:**	**40 h**							**Storage**	**42.708**	**717**	
**Registros:**	**155 of 155**										
**ID**	**Activity**	**Operations** 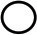	**Inspections** 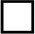	**Transport** 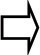	**Queue** 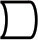	**Storage** 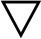	**Time required (hours)**	**Process inventory**	**Finished product** **(units)**	**Percentage** **(units)** **use**	**Constraints**
3	Check the biopsy request card data		x				2.336	239	0	5.84	
4	Number the biopsy consecutively	x					5.274	239	0	13.19	
5	Enter data into the SIPAT software	x					6.573	239	0	16.43	
7	Prepare formalin jars for the specimens	x					1.46	239	0	3.65	
8	Cut the specimens	x					4.621	239	0	11.55	
9	Enter the data into the SIPAT software	x					6.575	239	0	16.44	
10	Fix the specimens in formalin jars	x					1.997	239	0	4.99	
11	Send the specimens to histology			x			1.597	239	0	3.99	
12	Check the received specimens' information		x				2.34	239	0	5.85	
13	Centrifuge the jar contents	x					19.975	239	0	49.94	
14	Clean the embedder	x					0.399	239	0	1	
15	Translate the specimens			x			1.596	239	0	3.99	
16	Put the specimens in the containers					x	0.399	239	0	1	
17	Pedal-controlled paraffin-filling of the containers	x					0.799	239	0	2	
18	Cool the paraffin wax	x					0.399	239	0	1	
19	Store sample blocks in refrigerator					x	39.912	239	0	99.78	**x**
20	Clean microtome	x					0.399	239	0	1	
21	Put blade in microtome	x					0.399	239	0	1	
22	Translate the sample blocks to the microtome			x			1.596	239	0	3.99	
23	Put sample block in microtome	x					0.399	239	0	1	
24	Preliminary trim of the block	x					3.021	239	0	7.55	
25	Manual slicing	x					8.543	239	0	21.36	
26	Pass specimens to the bain marie	x					0.399	239	0	1	
27	Place tissue sample on the slide	x					0.399	239	0	1	
28	Number and dry the slide	x					19.968	239	0	49.92	
29	Place slides in basket					x	0.799	239	0	2	
30	Bring the basket over to the staining area			x			1.595	239	0	3.99	
31	Staining of the slides in the basket	x					4.488	239	0	11.22	
32	Storage of the slides					x	37.482	239	0	93.71	**x**
33	The pathologist goes to the slide storage area			x			36.95	239	0	92.38	**x**
34	The pathologist examines the slides	x					6.569	239	239	16.42	

### Economic Evaluation of the Proposals

Three proposals are made to reorganize the Anatomic Pathology Service. As shown in the diagnosis column, they are designed to eliminate the waits of up to 4 months that are currently the case, and to make the diagnoses in just 3 days. With these proposals, the current annual output of biopsy analyses would be raised from 4,850 to 8,300, which would represent a reduction in cost per analysis from US$10.64 to US$5.57. Given that the welfare of the service's user should be favored, it is recommended to implement proposal 3. Although it is not the most economical option in the short term, would be helping to deal with the backlog facing the pathology service, and afterwards the cost per biopsy would continue to be the same as proposals 1 and 2. Table 8 shows the cost benefit analysis for output plan proposals.

**Table 8:** Cost benefit analysis for output plan proposals.

**Proposal**	**Annual fixed**	**Overtime**	**Total**	**Adjustment**	**Annual**	**Diagnosis**	**Approx. cost**
	**cost (US$)**	**(US$)**	**cost (US$)**	**time (months)**	**output (units)**	**(days)**	**per biopsy (US$)**
1	46,225.00	0.00	46,225.00	NA	8,300.00	3	5.57
2	46,225.00	0.00	46,225.00	7.00	8,300.00	3	5.57
3	46,225.00	2,433.03	48,658.27	3.00	8,300.00	3	5.86
Current	23,112.62	28,518.61	51,631.23	NA	4,850.00	120	10.64

## Discussion

The tool developed is capable of representing the anatomic pathology process by means of a simulation model which incorporates such elements as this service's waiting times and biopsy throughput (34). By incorporating elements of variability and uncertainty through the Box-Muller algorithm, it was possible to identify those activities which represent bottlenecks in the process and limit its productive capacity with respect to the demand.

It was found that the processing times had a normal probability distribution. Nonetheless, this condition does not always prevail in processes, particularly in services where probability distributions tend to be skewed to the right, making it necessary to incorporate other types of algorithms (16, 38).

As part of the solutions, three proposals were determined which made it possible to evaluate aspects of an economic and operational nature. Each proposal has elements differentiating it from the others, among which there stands out the impact of considering the backlog of biopsies pending diagnosis by the pathologist (39, 40).

At a methodological level, the stages of continuous improvement of DMAIC processes were incorporated, focused on resolving problems of variability, in this case of the waiting times for the biopsy diagnoses. This structure is adequately integrated with the use of simulation in the analyse and improve stages. In the former case, this is to identify the main causes that affect the problem and the impact they have (41). In the latter case, it is to evaluate the implementation of the possible solutions, and thus choose that best suited to correcting the problem under study.

The improvement scenarios consider different assumptions to determine the behavior that they would have for the waiting time through the simulation model (42). The number and combination of scenarios could vary depending on the experience and training of the analyst (43). The following situations were defined in this study:

1. The first proposes a schedule under normal working conditions for two pathologists.
2. The second addresses also the number of biopsies that are pending diagnosis, normalizing the working conditions in 7 months by suspending autopsies for that period to focus on clearing the backlog.
3. The third is similar to the second but incorporates 320 overtime h for 3 months to finish the backlog of pending cytologies and biopsies during that time.

In subsequent studies, this analysis may be extended to cytologies and autopsies to reduce their waiting times. The results can be extrapolated to other health services (44, 45).

## Conclusions

A simulation model based on the Box-Muller algorithm has been developed with which it was possible to perform an analysis of the installed productive capacity to reduce the average waiting time for the diagnosis of clinical biopsies from 2 months to 3 days. With respect to the times for the diagnosis of biopsies by the pathologist, two groups of data were distinguished, one with a mean of 9.257 min and a standard deviation of 7.307 and another with a mean of 27.389 min and a standard deviation of 6.001, both with a normal distribution. The hypothesis that 2.5 biopsies are performed per hour (24 min per biopsy) was therefore rejected.

The model simulating the process was able to show that, with two pathologists, the pathology department can output about 8 000 biopsies per year. To meet this annual output, 315 cutting h and 1 697 diagnosis h would be needed. In the improvement scenarios, it was concluded that it is possible to diagnose a biopsy in 3 working days, as long as the usual conditions defined apply. It is necessary to take into account in the programming the number of biopsies that are pending diagnosis.

## Data Availability Statement

The raw data supporting the conclusions of this article will be made available by the authors, without undue reservation.

## Author Contributions

JG-S-C: conceptualization, supervision, and validation. FB-M: data curation, funding acquisition, and writing—original draft. FB-M, BV-V, and JG-S-C: investigation. BV-V and OV-A: methodology. OV-A: project administration, resources, and writing—review and editing. BV-V: software and visualization. All authors contributed to the article and approved the submitted version.

## Funding

This research was funded by the Vice Rectory for Research and Extension, Grant No. 5402-1351-2401, Design of a decision model for the organization of resources according to their availability by Simulating Discrete Events (2020–2021).

## Conflict of Interest

The authors declare that the research was conducted in the absence of any commercial or financial relationships that could be construed as a potential conflict of interest. The reviewer GS-B declared a shared affiliation with one of the authors JG-S-C to the handling editor at time of review.

## Publisher's Note

All claims expressed in this article are solely those of the authors and do not necessarily represent those of their affiliated organizations, or those of the publisher, the editors and the reviewers. Any product that may be evaluated in this article, or claim that may be made by its manufacturer, is not guaranteed or endorsed by the publisher.
